# Forces Controlling the Assembly of Particles at Fluid Interfaces

**DOI:** 10.1021/acs.langmuir.2c02038

**Published:** 2022-10-24

**Authors:** Eduardo Guzmán, Francisco Ortega, Ramón G. Rubio

**Affiliations:** † Departamento de Química Física, Facultad de Ciencias Químicas, Universidad Complutense de Madrid, Ciudad Universitaria s/n, 28040Madrid, Spain; ‡ Instituto Pluridisciplinar, Universidad Complutense de Madrid, Paseo Juan XXIII 1, 28040Madrid, Spain

## Abstract

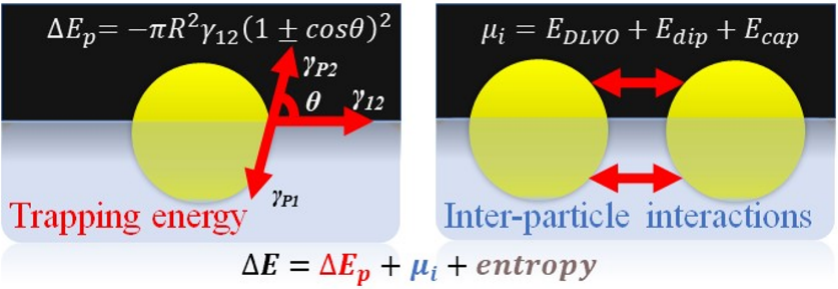

The interaction of particles with fluid interfaces is ubiquitous in synthetic and natural work, involving two types of interactions: particle–interface interactions (trapping energy) and interparticle interactions. Therefore, it is urgent to gain a deep understanding of the main forces controlling the trapping of particles at fluid interfaces, and their assembly to generate a broad range of structures characterized by different degrees of order. This Perspective tries to provide an overview of the main contributions to the energetic landscape controlling the assembly of particles at fluid interfaces, which is essential for exploiting this type of interfacial systems as platforms for the fabrication of interface-based soft materials with technological interest.

## Introduction

Studies on the stabilization of emulsions by Ramsden and Pickering at the dawn of the 20th century were the pioneering works opening a research field that has undergone strong development in the last few decades.^1,2^ Indeed, particle-laden fluid interfaces are very interesting models for studying different aspects of the physics of quasi-2D systems, including their phase diagrams and interparticle interaction potentials.^3^ On the other hand, particle-laden interfaces also offer a versatile platform for the fabrication of a broad range of interface-dominated soft materials, e.g., colloidosomes and capsules, photonic crystals, integrated circuits, and many other.^4^ Moreover, the stabilization of interfaces by particles is essential to stabilizing new ecofriendly emulsions and foams, avoiding the use of surfactants, which can be a very important advantage for industry.^5^

The multiple perspectives offered by the study of particle-laden interfaces are due, in part, to the broad range of systems that can be designed by the assembly of particles at fluid interfaces. This has been stimulated by the strong development of synthetic routes, which enable the controlled fabrication of particles differing in their shapes, sizes (from a few nanometers to several micrometers), or surface chemistries. This has paved the way for modulating, almost at will, the particle physicochemical and structural characteristics and hence their interactions in the bulk and upon their attachment to fluid interfaces.^6,7^ This enormously broadens the phenomena arising upon the adsorption of particles at fluid interfaces, allowing the preparation of particle-laden fluid interfaces with different equilibrium and dynamic properties modulated by different interaction potentials.^3,8^ In fact, particle-laden fluid interfaces offer an excellent platform for the fabrication of all-liquid materials characterized by the confinement of the particles in specific geometries.^9,10^

In this Perspective, we will briefly highlight the current understanding on the most fundamental physicochemical aspects underlying the assembly of particle-laden interfaces. This requires us to consider two different types of forces: (i) forces guiding the trapping of the particles to the fluid interface and (ii) interparticle interactions operating between particles after adsorption. An effective quantification of the forces driving the particles from the bulk to the interface and those controlling the interaction between particles once they are trapped at the interface is of paramount importance. Indeed, it is imperative for deepening on the understanding of the equilibrium and dynamic behavior of particles trapped at fluid interfaces as well as for exploiting this type of systems beyond academia.

## Guiding the Trapping of Particles to the Interface: Interfacial Tension and Contact Angle

The accumulation of particles at fluid interfaces, as occurs for the segregation of molecular species, is mediated by the reduction of the surface excess Gibbs energy which can be accounted by the interfacial tension, γ_12_, between the two immiscible fluids and those of each of the fluids with the solid surface of the particles. This can be understood by considering that the accumulation of particles at the interface is associated with the reduction of the contact area between the two fluids due to the rupture of the interface continuity. This contact area reduction is accompanied by a decrease in the free energy of the system. However, since the particle size exceeds, in most of the cases, the interface thickness, it is not possible to apply the classical microscopic definition of the interfacial tension to describe its change as a result of the formation of the particle-laden fluid interface.^5^ Therefore, a standard thermodynamic description of the interfacial tension of particle-laden fluid interfaces is not possible, making it necessary to consider the interfacial tension as an effective magnitude. This is a very important drawback to the development of a physically reliable thermodynamic description of particle-laden interfaces. To date, most models provide only a phenomenological description of the thermodynamic behavior of particle-laden, which is, in many cases, incomplete.^7^

The adsorption of particles to the fluid interface creates a 2D lateral pressure Π opposing the reduction of the interface area guided by the interfacial tension, which in turn forces the interfacial tension to decrease. This is the result of a complex interplay between entropic contributions and interparticle interactions, allowing an interfacial tension for the particle-laden interface defined as γ = γ_12_ – Π. The lateral pressure contribution is associated with the interfacial coverage (Γ) and the energy associated with the trapping of a single particle to the fluid interface (Δ*E*_p_), according to Π(Γ) = Γ|Δ*E*_p_*|.*^10^

**Figure 1:**
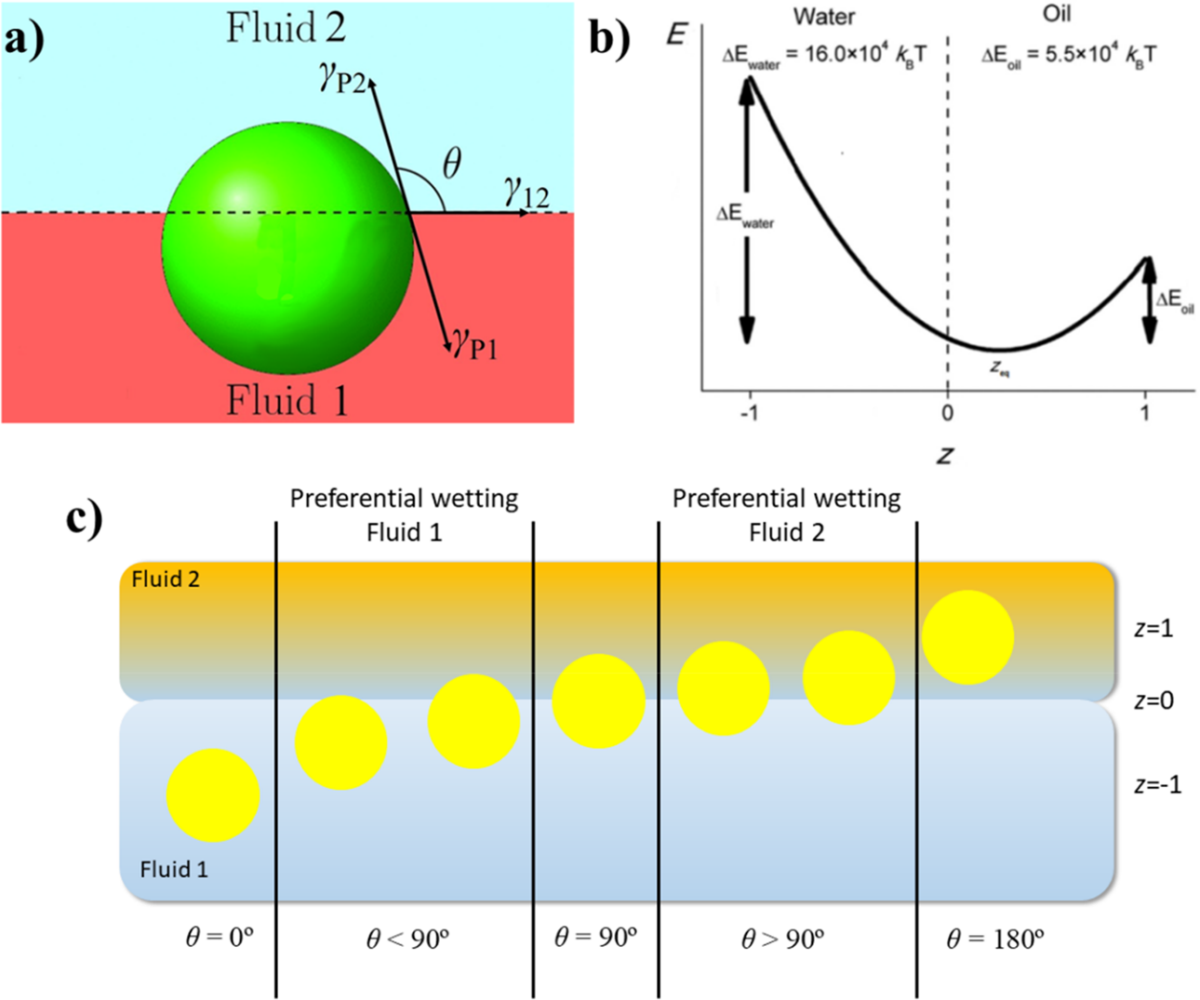
(a) Sketch of the mechanical equilibrium for a particle trapped at a fluid interface. θ represents the particle contact angle, *γ*_P1_ and *γ*_P2_ are the interfacial tensions between the particle and the two fluid phases, and *γ*_12_ is the interfacial tension corresponding to the fluid interface. Adapted with permission from ref (13). Copyright (2014) American Institute of Physics. (b) Evolution of the energy of a particle with its position with respect to the interfacial plane (*z*). The minimum in the potential energy curve at *z*_eq_ indicates the equilibrium position of the particle upon its trapping at the fluid interface. The detachment energies associated with the transport of the particle from the interface to the oil (Δ*E*_oil_) or water (Δ*E*_water_) phases are indicated in the panel. Adapted with permission from ref (15). Copyright (2019) Royal Society of Chemistry. (c) Evolution of the contact angle of a particle as a function of its position with respect to the interfacial plane.

Beyond the decrease in the interfacial tension, the change in energy associated with the trapping of particles to the fluid interface plays a central role in the final equilibrium state of a particle-laden interface. Δ*E*_p_ = *E*_interface_ – *E*_bulk_, where *E*_interface_ and *E*_bulk_ are the energies of a particle at the fluid interface and dispersed in the continuous phase, accounting for the change in energy associated with the transference of a particle from a dispersion to its equilibrium position at the fluid interface.^11,12^ This change in energy characterizes the mechanical equilibrium conditions of the particle-laden interface, which can be defined in terms of Young’s law according to the geometrical description sketched in Figure 1a.^3,13^ In fact, considering that the sum of the different forces (interfacial tensions) operating within the interfaces takes a null value (0 = *γ*_P__1_ – *γ*_P__2_ – γ_12_ cos θ, with γ_P__1_ and γ_P__2_ being the solid/fluid interfacial tensions between the particle and fluids 1 and 2, respectively, and θ being the particle contact angle with the interface), it is possible to provide a definition of Δ*E*_p_ for the trapping of a spherical particle to an interface between two immiscible fluids, according to the following relationship^14^
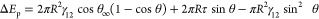
1where *R* accounts for the particle radius and τ is the line tension. θ_∞_ is the macroscopic contact angle, which defines the mechanical equilibrium conditions for an infinitely large particle trapped at the fluid interface. This may be considered equivalent to the contact angle of a liquid droplet with a molecularly smooth flat surface presenting surface chemistry similar to that of the particle. Thus, it is possible to define a modified Young’s equation as
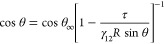
2In general, except for very small particles or when their roughness is very high, the role of the line tension can be neglected and cos θ = cos θ_∞_. This simplifies the definition of the trapping energy, which can be defined as
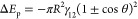
3The ± sign considers that particles can present different affinities for the fluid phases, and hence the mechanical equilibrium conditions are not defined, in most cases, by an equal immersion of the particles in both fluid phases. In other words, the equilibrium position of the particle center with respect to the interfacial plane *z*_eq_ does not assume a null value (*z* = 0 accounts for the position of the interfacial plane, whereas *z* = −1 and 1 indicate the bottom and upper phases), leading to two boundary situations. The first one corresponds to the particle center being placed above the interfacial plane (i.e., particles are mostly immersed in the upper phase, generally the most nonpolar one), and the + sign applies. The second limit considers that the particles are placed with their centers below the interfacial plane (i.e., particle are mostly immersed in the bottom phase, generally the most polar one), and the – sign has to be used.^15^ This reads as two well-differentiated regions for the relative wettability of particles for the fluid interface, with θ = 90° defining the border between such regions (equal immersion of the particle in both fluid phases, *z*_eq_ = 0).

Figure 1b shows an idealized energetic landscape characterized by the total energy *E* for the trapping of a hydrophobic particle at an arbitrary interface between polar and nonpolar phases (e.g., the water/oil interface) as a function of the height coordinate (*z*). It should be noted that the lower the detachment energy of a particle to one of the fluid phases (−Δ*E*_p_, in Figure 1b defined as Δ*E*_oil_ and Δ*E*_water_), the higher the affinity of the particle for such phase (i.e., *z*_eq_ ≠ 0, with *z*_eq_ being the equilibrium position of the particles in relation to the interfacial plane). Thus, for the particular case of Δ*E*_oil_ < Δ*E*_water_ represented in Figure 1b, a higher penetration of the particle into the oil phase should be expected. Indeed, *z*_eq_ corresponds to the height coordinate at which the free energy is minimized (∂*E*/∂*z* = 0) and depends on the three interfacial tensions and the particle size () as well as on the particle hydrophilic–lipophilic balance (HLB) determined by θ. Thus, for an interface between two fluids with very different values of their dielectric constants, hydrophilic particles are placed with their centers below the position *z* = 0 (i.e., θ < 90°), whereas hydrophobic particles are mostly immersed in the upper phase with θ > 90°. Figure 1c shows the evolution of the particle position with respect to the interface as a function of its contact angle. It should be stressed that the position of the particle in relation to the interfacial plane plays a major role in the interface stabilization, which is critical for the stabilization of Pickering emulsions and foams.^16^ This may be altered by additives (e.g., surfactants, polymers, or small molecules) which allow a reversibly or irreversibly modification of the chemical nature of the particle surface in addition of a modification of the interfacial tension and hence alter its relative wettability for the interface (i.e., change the particle contact angle).^17^

**Figure 2:**
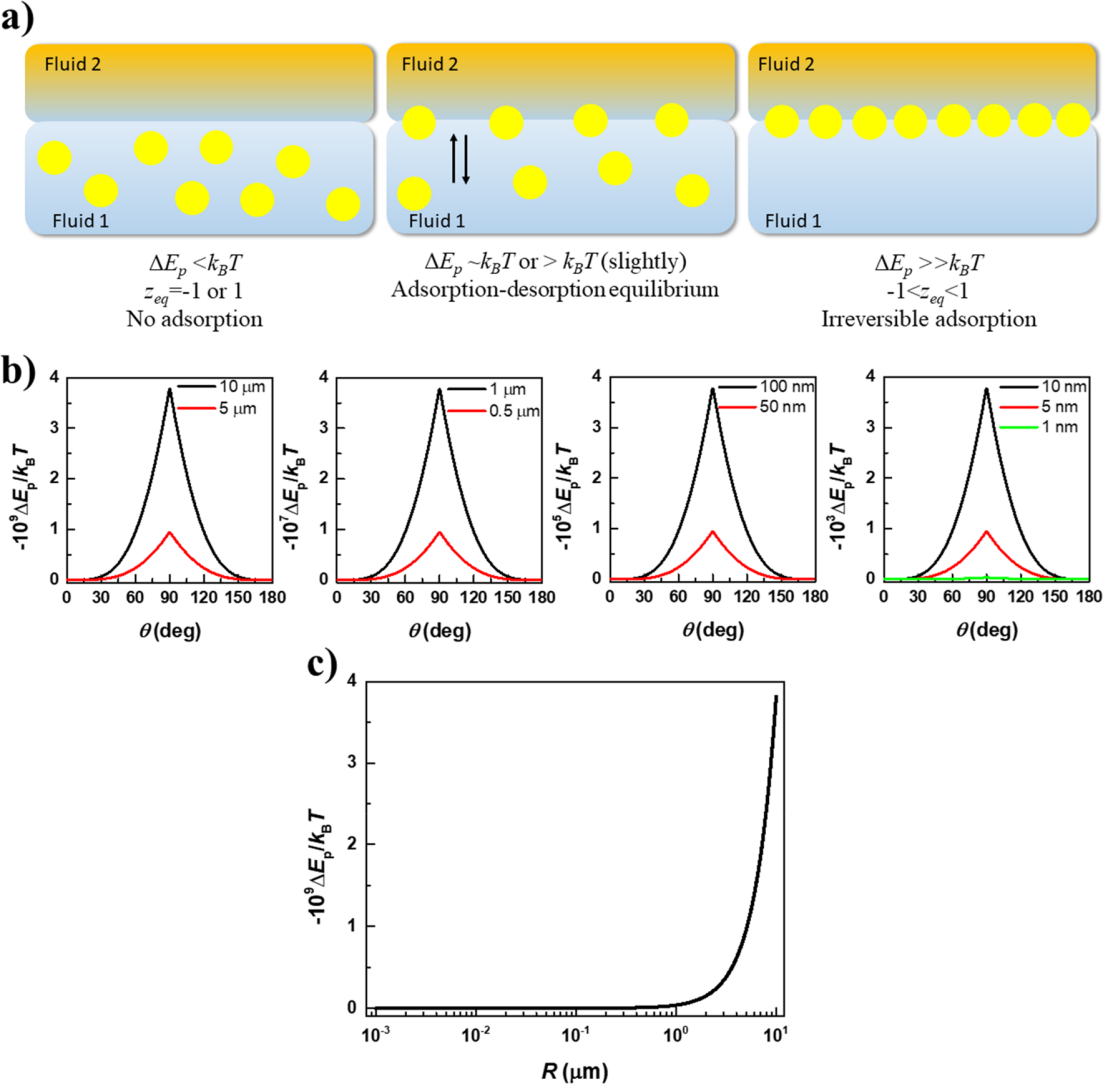
(a) Sketch of the three different states that can appear for the interaction of particles with fluid interfaces as a function of the value of Δ*E*_p_. (b) Contact angle dependence of the trapping energy of colloidal particles with different values of *R* at a fluid interface with *γ*_12_ = 50 mN/m at 25 °C. Notice the different values of the scales. (c) Dependence of the trapping energy for a contact angle of 90° on the particle radius for an interface with *γ*_12_ = 50 mN/m at 25 °C.

The above framework is valid only when the capillary binding of the particles to the fluid interface is strong enough to avoid the detachment phenomena associated with the thermal fluctuations. This means that the trapping of particles to a fluid interface requires that Δ*E*_p_ exceeds several times the thermal energy (*k*_B_*T*, where *k*_B_ is the Boltzmann constant and *T* is the absolute temperature). Otherwise, the trapping of the particles does not occur (Δ*E*_p_< *k*_B_*T*) and particles are expelled to one of the fluid phases (*z*_eq_ = −1 for the aqueous phase and 1 for the oil phase), or if it occurs, it can be associated with an adsorption–desorption equilibrium as occurs in molecular surfactants (Δ*E*_p_ ≈ *k*_B_*T* or only slightly higher than *k*_B_*T*).^18^Figure 2a shows three sketches representing the different situations that can occur for the trapping of particles at a fluid interface depending on the value of Δ*E*_p_.

The strength of the particle trapping to the fluid interface is modulated by three different parameters: θ, *R*, and *γ*_12_. The former provides information on the relative wettability of the particle for the interface and can be modified by the chemical nature of the particles and their physicochemical properties (e.g., roughness, size, and shape), and *γ*_12_ depends only on the physicochemical properties of the fluids forming the interface. Therefore, if we consider a particular case in which spherical particles characterized by the same surface roughness and the same wettability for an interface formed by the same immiscible fluids adsorb to the interface, then the tendency of the particles to remain trapped at the fluid interface will depend only on the particle size (eq 1).

Figure 2b shows the dependence of Δ*E*_p_ on the contact angle for the trapping of spherical particles with different values of *R* to an arbitrary interface with *γ*_12_ = 50 mN/m. The trapping energy takes negative values for particles with diameters ranging from several micrometers to 1 nm. However, from the plots in Figure 2b, it is clear that the strength of the particle binding to the interface is strongly dependent on the particle dimension. This may be better understood from the dependence of the trapping energy for particles with a contact angle of 90° on the particle dimensions depicted in Figure 2c. The adsorption of micrometer-sized particles to a fluid interface is associated with trapping energies that largely exceed the thermal energy *k*_B_*T*. Thus, the adsorption of micrometer-sized particles is irreversible except for very low values of the *γ*_12_ and/or for cos θ → 1. It should be noted that the trapping of particles to the fluid interface is a dynamic process characterized by continuous aging with time.^19^ This allows understanding because particles trapped with a very small contact angle to the interface can desorb by considering that during their progress toward their equilibrium position they can pass through configurations characterized by a binding energy similar to the thermal energy, and hence a small proportion of microparticles can undergo a spontaneous desorption from the interface.^3^ However, this is not the common picture appearing for most of the experimental systems.^11^ On the other side, as the particle size tends to the nanometric scale, the trapping energy decreases to values on the order of several *k*_B_*T*, which can drive the thermally activated escape of the particles from the interface as occurs in molecular surfactants, polymers, and proteins.^3^

The above framework can be applied only when the size of the particles is small enough to ensure that the interfacial tension can overcome the gravity-driven sedimentation. This requires that the Eötvos number , where *Δρ* denotes the density difference between the solid particle and the fluid and *g* is the acceleration due to gravity, remains well below 1 as occurs for particles smaller than 10 μm, and the interfacial tension forces are the dominant contribution, except for heavy particles in which gravity may play a non-negligible role.^13^

The above description of the trapping of particles to fluid interfaces is a cost-effective approach for evaluating the adsorption of spherical colloids at fluid interfaces in terms of three parameters: *R*, *γ*_12_, and θ. The former two are easily accessible experimentally, whereas the evaluation of the contact angle is not always trivial, even though there are currently a broad range of techniques providing information on such a parameter. However, the contact angle values provided by the different techniques is not always the same, which makes it difficult to know its real value.^3,12^ On the other side, there are several aspects that may modify the energetic landscape associated with the trapping of particles to fluid interfaces. These include the shape and size of the particles, the roughness of the particle surface, and the pinning–depinning phenomena of the contact line. These contributions are commonly included within the line tension contribution, which modifies the trapping energy (eq 1).^11^ Moreover, the situation becomes even more complex for curve interfaces, where the presence of curvature modifies three different aspects influencing the trapping energy: (i) the Laplace pressure, (ii) the interfacial profile, which becomes more complex; and (iii) the contact line. This leads to a curvature-induced lateral force that tends to expel the particle from the interface, reducing the trapping energy.^20^ Similarly, particle anisotropy influences the binding of the particles to the interface. In fact, anisotropy can lead to the adsorption of particles with a certain tilt angle that modifies the penetration of the particles into the interface, which in turn affects the trapping energy.^21^

## Controlling the Assembly of Particles at the Interface: Interparticle Interactions

**Figure 3:**
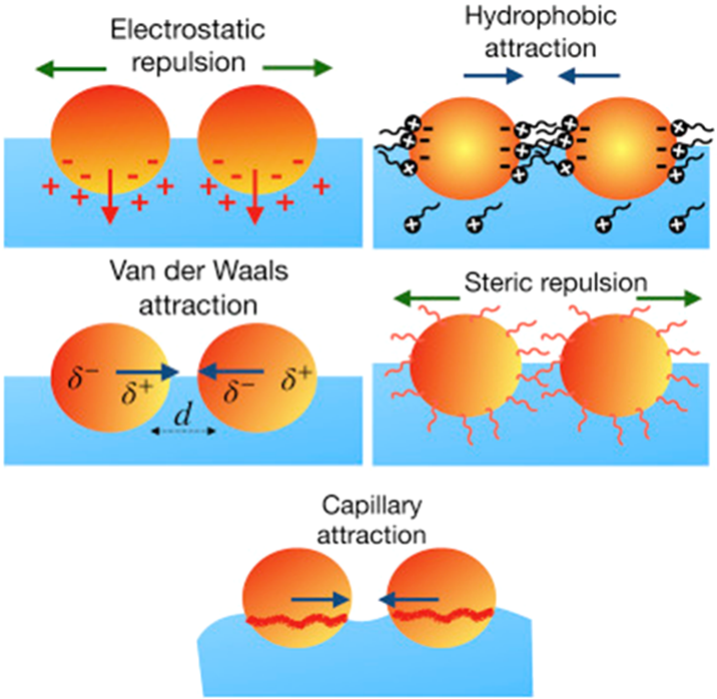
Idealized pictures representing some of the most common interparticle interactions appearing in particle-laden interfaces. Adapted with permission from ref (23). Copyright (2019) Elsevier.

Once the interface is covered by several particles, a broad range of interactions, with different origins, starts to operate between them.^22−24^Figure 3 shows a sketch representing some of the most common interactions appearing for particles trapped at fluid interfaces. Notice that the schemes consider particles with smooth surfaces and a homogeneous charge distribution, which can be far from the true situation, and introduce a modification to the importance and strength of the interparticle interactions occurring within the interface.^24^

A rigorous analysis of the pair interparticle interactions, in the absence of any external actuation, requires the consideration of two main groups of interactions: (i) direct interactions and (ii) interface-mediated interactions. The former one also appear in bulk systems, even though they are slightly modified by the presence of the interface, whereas the second type of forces emerges as a result of the confinement at the interface (capillary and hydrodynamic interactions), and cannot be found in the absence of the fluid interface. The latter are strongly dependent on the specific chemical and morphological characteristics of the particles as well as on the nature of the fluid phases involved in the interface.^24^ Following an analogous method used for the trapping energy, it is possible to define the balance of interparticle interactions involved in the assembly of particles at a fluid interface in terms of the difference between the energy associated with the particles at the interface, *E*_int_, and that corresponding to the particles dispersed in the bulk, *E*_bulk_:
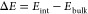
4As stated above, a rigorous evaluation of the free-energy change Δ*E* associated with the assembly of a particle-laden interface implies the consideration of several contributions, including the trapping energy, Δ*E*_p_, the balance of interparticle interactions (including van der Waals, electrostatic, capillary, density fluctuations, or hydrophobic ones) operating within the interface, *μ*_i_, and the unfavorable entropic contribution, *μ*_e_, associated with the decrease in the translational degrees of freedom of the particles once they attach to the interface. Therefore, it is possible to define the free-energy change associated with the formation of a particle-laden interface according to the following general expression:
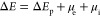
5It should be stressed that while the contribution associated with the trapping energy considers individual particles, the contributions associated with the entropy and interparticle interactions emerge from the particle coupling. Therefore, they depend on the interparticle distance and hence are modulated by the interfacial coverage and the volume fraction of particles in the bulk. This is of paramount importance because the properties of particle assemblies at fluid interfaces can be manipulated through the modification of the interparticle interaction potentials, the particle characteristics (roughness and shape), the temperature, or the particle tendency to remain trapped at the fluid interface.^9^

### Direct Interactions

The first direct forces are the van der Waals interactions which account for the interaction of the different components of the particle. This type of interaction also appears in the particle dispersion, even though its contribution is weaker. Despite the importance of the van der Waals interactions for the energetic landscape of particle-laden interfaces, their quantification is not trivial, and only an effective van der Waals contribution can be obtained by introducing an effective Hamaker constant of the particle across the two fluid phases and the fraction of particle immersed in fluid 2 (i.e., *z*_eq_). Therefore, the effective van der Waals contribution between a pair of particles trapped at an arbitrary fluid interface depends on the specific nature of the particles and the fluid phase as well as on the relative wettability of the particle for the interface, thus linking the van der Waals contribution to the trapping energy.

The contribution of the electrostatic interactions differs from that appearing in bulk systems because the presence of the interface adds to the short-range screened Coulomb component, reminiscent of the bulk behavior, which is a long-range dipole–dipole interaction. This latter is strongly dependent on the specific nature of the fluid phases. It should be noted that the screened Coulomb component can be neglected when the interfacial coverage is very low.

The confinement associated with the interface introduces asymmetric character into the electrostatic interparticle interactions. In fact, the dipolar contribution for particles at a water/nonpolar fluid interface occurs very differently across the water and nonpolar phase due to the differences in the electrical permittivity of each of the phases.^25^ For the case of the particle fraction immersed, the interactions occur between the dipoles formed by the dissociated surface groups of the particles and the free counterions existing in the bulk, whereas the interaction across the nonpolar phase occurs between the particle surface charges and the image charges appearing in the water.^24^ Therefore, the position of the particles in relation to the interfacial plane (i.e., *z*_eq_) also plays a central role in the strength of the dipole–dipole repulsion. Moreover, the strength of the electrostatic contribution is also influence by the degree of screening of the interactions across the aqueous phase. It should be noted that when particles with rough surfaces are considered, water with ions could be taken from the aqueous phase to the nonpolar one, thus modifying the dipole–dipole interactions.^26^ Finally, the role of the double-layer contribution to the well-known DLVO, which is strictly valid for two flat layers much larger than the ions in the solution, may not be acceptable when particles trapped at fluid interfaces are considered.^27^

Other direct interactions contributing to the energetic landscape of particle-laden interfaces, even though their role is less important than that corresponding to van der Waals and electrostatic ones, are the hydrophobic and steric ones. The former emerges from the necessity to minimize the unfavorable contacts between a hydrophobic surface and water, depending of the molecular details of both the particles and fluid phases. However, there is no any systematic quantification of the role of the hydrophobic interactions in particle-laden interfaces.

The steric interactions emerge from the presence of additives in particle dispersions to modify their stability by the formation of a capping layer on the particle surface. The presence of the capping layer leads to osmotic pressure as particles approach each other. This creates an unfavorable entropic contribution associated with the compression of the capping layer, resulting in a repulsive contribution to the energetic balance of the system.

The existence of direct interactions introduces a contribution to the energetic landscape of particle-laden interfaces, which can be approached as a combination between a DLVO component (*E*_DLVO_), including the van der Waals forces and the long-range Coulombic interactions, and a short-range dipolar interaction (*E*_dipolar_). These interactions are closely correlated to the equilibrium position of the particles at the fluid interface. The modification of the direct interactions as the interfacial coverage is changed is a very important issue from a material perspective because it drives the formation of particle assemblies with a broad range of structures that control the mechanical performance of the interface, providing different degrees of stability to the interface.^28^

### Interactions Mediated by the Presence of an Interface

The interfacial confinement also introduces new interparticle forces which are not found in bulk systems. The most important contribution is due to the capillary forces, which are the result of the deformation of the interface due to the particle adsorption.^29^ This is strongly dependent on the properties of the particles. In particular, the size and density of the particles dominate the deformation of the interface. For instance, the gravity effects induced by a large particle produces a strong deformation of the interface, which is translated into the appearance of the flotation forces, governed by different parameters, including particle shape, buoyancy, and trapping energy of the particles at the fluid interface. The reduction of the particle size decreases the strength of the flotation forces down to reach null values for particles smaller than the capillary length of the fluid interface.

Despite the fact that the contribution of the flotation forces is reduced with the particle size, this does not mean that the capillary contribution must be neglected in the energetic balance. Thus, even though the weight of the particles cannot be enough to deform the interface, local deformations of the particle contact line can appear, which result in long-range capillary immersion forces. Their strength is modulated by the roughness and chemical heterogeneity of the particle surface, which contribute to the formation of nonsmooth, wavy, or irregularly shaped three-phase contact lines.^24,30^

The presence of the interface can also create hydrodynamic interactions which are associated with the motion of the particles through a viscous medium. This motion of the particles creates a flow which can be modified for the rest of particles at the interface, creating an interface-mediated interaction. However, the role of the hydrodynamic interactions is not always to quantify, playing a more minor role in the energetic landscape than the capillary interactions.

**Figure 4:**
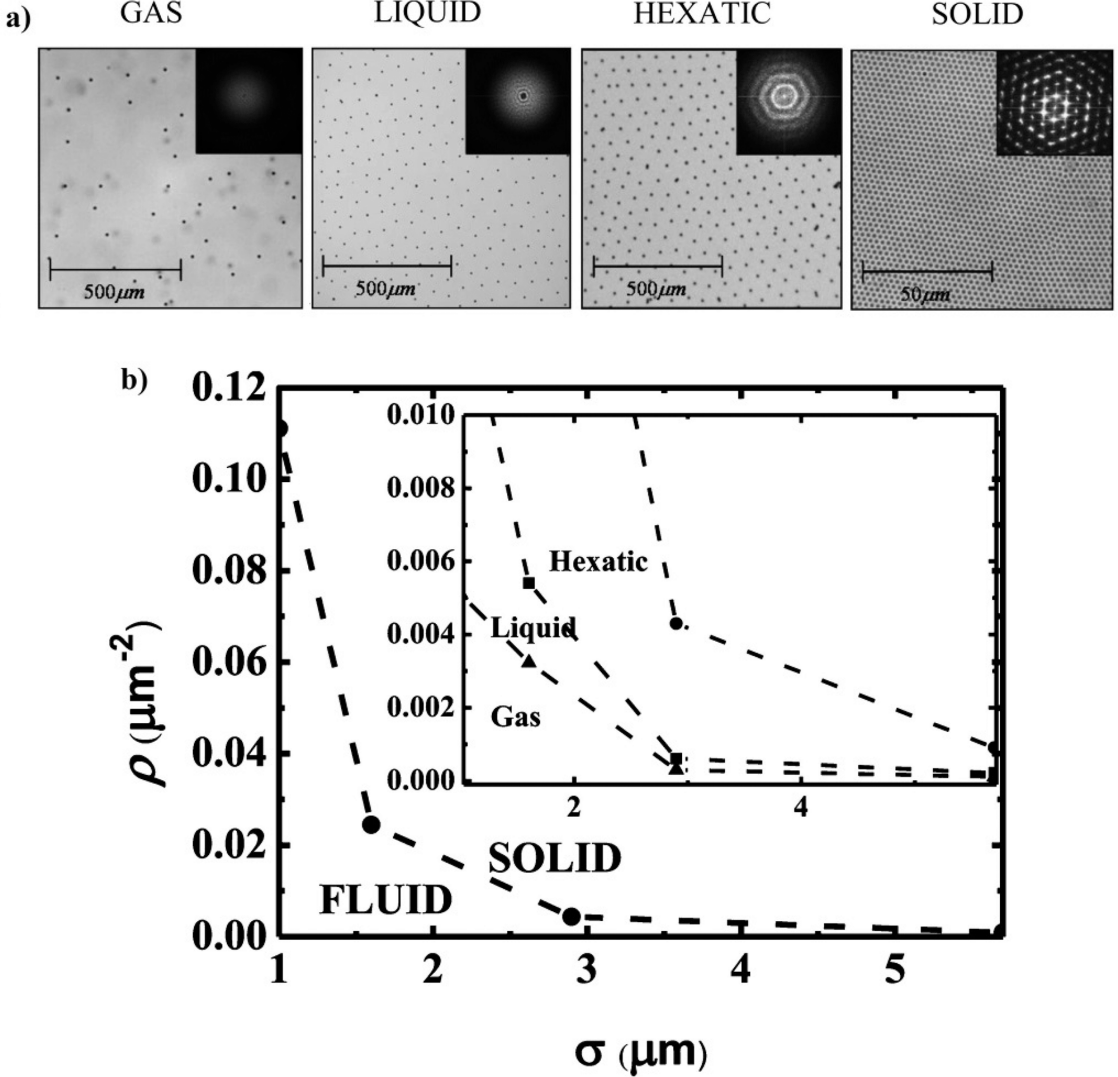
(a) Optical microscopy images for particle-laden water/octane interfaces at different interfacial coverages (representing different phases) and their corresponding FTT images (insert panels). (b) Phase diagram of a particle trapped at a water/octane interface. Adapted with permission from ref (32). Copyright (2011) American Chemical Society.

According to the above discussion, it may be possible to define that the interparticle interactions in particle-laden interfaces can be approximated by a combination of the contribution of the direct forces with the capillary contribution (*E*_capillary_), which can be summarized in the following expression:
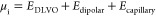
6The evaluation of the exact magnitude and ratio between the different contributions to the energetic balance of particle-laden interfaces requires a careful examination by using optical tweezers or other related techniques.^31^ This is of a paramount importance because it plays an essential role in controlling how particles assemble at the fluid interface, with the role of each contribution being very different depending on the interfacial coverage. (Figure 4a shows the change in the organization of particles at fluid interfaces depending on the interfacial coverage.) At very low values of the interfacial coverage, particles interact mainly through dipolar interactions. However, the increase in the interfacial coverage increases the importance of the attractive component, with the capillary force playing a prominent role in the final energetic landscape of particle-laden fluid interfaces. In fact, the presence of the strong capillary interactions plays an essential role in the ordering transition of particles at fluid interfaces as the interfacial coverage increases. (Figure 4b displays the phase diagram of polystyrene particles trapped at a water/octane interface as a representation of the dependence of the interfacial density of the transition, ρ, on the particle diameter, σ.^32^) The appearance of a strong attractive contribution as the density increases also affects the dynamics of particles trapped at the fluid interface. In fact, at low values of the interfacial coverage, particles at the fluid interface describe a motion that can be interpreted in terms of Brownian dynamics, which becomes subdiffusive as the interparticle interactions start to operate within the interface (i.e., the motion becomes slower as the interfacial coverage is increased).^33^

It should be stressed that the control of the interparticle interaction is essential to tuning the particle organization at the interface, which in turn may alter the mechanical properties of the interfaces. This becomes a very important issue when the assembly of particles on oil droplets or gas bubbles in emulsions and foams is considered.^34^

## Summary

This Perspective has provided a general approach to the balance of interactions governing the assembly of particle-laden interfaces. These can be divided into two contributions: the first one is related to the driving force guiding the trapping of the particles from the bulk dispersion to the interface, and the second one is associated with the forces that start to operate between particles once they reach their equilibrium position at the interface. The latter is, in part, reminiscent of that which occurs in bulk dispersions, including some additional contributions arising from the confinement effects induced by the presence of the interface. Despite the fact that the two energetic contributions drive the equilibration of particle assemblies at fluid interfaces, they cannot be separated. In fact, the interfacial interparticle interactions are strongly affected, among other parameters, by the volume fraction of the particle immersed in each fluid. This depends on the equilibrium position of the particle with respect to the interfacial plane and hence on the relative wettability of the particles with respect to the fluid interface. Therefore, it may be expected that the strength of the interparticle interactions can be influenced by the strength of the binding of the particles to the fluid interface.

The above discussion has addressed the description of the simplest case considering the assembly of spherical colloids at fluid interfaces. However, it provides a framework allowing the understanding of the driving forces guiding the assembly process of particle-laden interfaces independently of the dimensions and shape of the particles. This is important because the control of the interparticle interactions becomes a critical issue in tuning the 2D organization of particles at fluid interfaces, which is essential for the mechanical performance of particle-laden interfaces and hence for their potential exploitation as platforms for building interfacial-based soft materials with technological interest.
